# A Cone Beam Computed Tomography-Based Investigation of the Frequency and Pattern of Radix Entomolaris in the Saudi Arabian Population

**DOI:** 10.3390/medicina59112025

**Published:** 2023-11-17

**Authors:** Muhammad Qasim Javed, Swati Srivastava, Badi Baen Rashed Alotaibi, Usman Anwer Bhatti, Ayman M. Abulhamael, Syed Rashid Habib

**Affiliations:** 1 Department of Conservative Dental Sciences, College of Dentistry, Qassim University, P.O. Box 1162, Buraidah 51452, Qassim, Saudi Arabia; s.kumar@qu.edu.sa (S.S.); bb.alotaibi@qu.edu.sa (B.B.R.A.); 2 Department of Operative Dentistry, Islamic International Dental College, Riphah International University, Islamabad 44000, Pakistan; usman.anwer@riphah.edu.pk; 3 Department of Endodontics, Faculty of Dentistry, King Abdulaziz University, P.O. Box 80209, Jeddah 21589, Saudi Arabia; amahmad4@kau.edu.sa; 4 Department of Prosthetic Dental Sciences, College of Dentistry, King Saud University, P.O. Box 60169, Riyadh 11545, Saudi Arabia

**Keywords:** cone beam computed tomography, cross-sectional studies, diagnostic imaging, dentistry, endodontics, mandibular molars, prevalence, radix entomolaris, root canal anatomy, three-dimensional imaging

## Abstract

*Background and Objectives*: An understanding of the anatomical complexity of teeth is a significant factor for a successful endodontic treatment outcome. The aim of this study was to explore the frequency and pattern of distribution of radix entomolaris (RE) in mandibular first molars (MFMs) of a Saudi Arabian subpopulation using CBCT scans. *Materials and Methods*: This study was conducted at dental clinics of Qassim University from February to May 2023 by evaluating CBCT scans that were previously obtained for diagnostic purposes. Scans of Saudi national patients with bilaterally present MFMs and fully formed root apices were included. Conversely, scans with one/or two missing MFMs, MFMs with incomplete root apices, full- or partial-coverage prosthesis, endodontic treatment, and associated radicular resorption were excluded from study. A total of 303 CBCT scans with 606 bilateral MFMs were analyzed by two calibrated evaluators for the presence of, and type according to Song’s typolgy of RE. The data were analyzed using SPPS-24. The descriptive variables were documented as frequencies and percentages. The chi-square test was used to determine the association between the prevalence of RE with the gender, jaw side and age group. Both inter-rater and intra-rater agreements were estimated for detecting and classifying RE using Cohen’s kappa test. *Results*: The sample had 63.7% males and 36.3% females. The prevalence of RE was 6.6%, with Song’s type III (57.5%) as the most common variant. Absolute agreement was noted between the raters about the presence of RE and very strong agreement was noted for the classification of the RE. *Conclusions*: RE is an uncommon finding among the mandibular first molars of the Saudi population without any gender and quadrant predilection. The clinicians’ knowledge of the presence and Song’s type of RE may contribute towards the enhancement of endodontic treatment outcomes.

## 1. Introduction

Successful endodontic treatment (ET) relies on various factors, including an anatomic understanding of the teeth and root canal system (RCS), which serves as the anatomical foundation for ET [1,2]. Failing to identify a single root canal during ET can result in the development of secondary or persistent apical periodontitis (AP) [3]. The first permanent teeth to appear in the oral cavity are the mandibular first molars (MFMs). These teeth, often referred to as the “key of the occlusion”, possess various pits and fissures on the occlusal surface, making self-cleaning a challenging process. As a result, individuals with a higher susceptibility to tooth decay are more prone to requiring ET for MFMs [4,5,6].

The cleaning and shaping of the RCS in the MFMs pose a great challenge due to the unpredictability of its morphological characteristics [7,8,9]. The typical shape of MFMs is described as having two roots and two canals in the mesial root, with one or two canals in the distal root [1]. Nevertheless, there are various deviations in the canal structure of mandibular molars, one being the presence of an extra distolingual root and canal [10], as well as the existence of a third canal in the mesial root known as the middle mesial canal [11]. The existence of additional root and complex root anatomy holds clinical significance as it can impact various dental procedures, such as prosthetic restorations, apical surgery, extraction, intentional replantation, and ET [12,13].

The understanding of the anatomical complexity of mandibular molars has improved over time. Modern imaging techniques like cone beam computed tomography (CBCT) have substantially increased the identification of various morphological variations like accessory middle mesial canals, distolingual roots, and distobuccal roots [14,15,16,17]. The existence of an additional root, referred to as radix entomolaris, at the distolingual aspect is an important variation. While this extra root has been associated with a notable ethnic characteristic in Asian countries, its prevalence in other regions of the world remains uncertain [7]. The accessory distolingual root was first discovered by Carabelli in 1844 [18]. It was later named radix entomolaris (RE) by Bolk in 1915 [19]. Typically, RE is characterized by its curvature and relatively shorter length compared to other roots, and may either be fused or separate from the main distal root [20]. Generally, RE is shorter than the distobuccal root, but in some cases, it may be completely separate from the DB root or fused with it [21]. Such anatomic nuances pose a serious challenge in root canal treatment for clinicians. For instance, the orifice of the RE is hard to locate without an appropriate modification of the access cavity, and when left untreated, these canals become a major cause of post-treatment disease due to microbial contamination [22,23]. Thus, a knowledge of the regional prevalence of distolingual roots is of great clinical significance to indigenous clinicians.

The presence of RE is a variable occurrence among different populations. A recent meta-analysis concluded a 5.6% global prevalence of this peculiar anatomic feature [24]. Studies of certain East Asian ethnic groups report a frequency as high as 32% [7]. In contrast, there are reports of zero percent prevalence in other ethnicities [14]. Several theories try to explain the etiology and the race-related high prevalence of this trait [14,16]. One such idea is the possible hereditary role of these three-rooted mandibular first molars, but this is still debatable due to a lack of adequate evidence [14,15,16,17,18,19].

Among the Saudi population, there is a paucity of CBCT-based evidence on the distribution of RE. Al-Alawi and colleagues reported a 4.3% presence of RE, while Mashyakhy et al. described a 2.9% prevalence [25,26]. The difference in the reports and the lack of high-quality CBCT-based studies warrant further investigation of this anomaly. Moreover, this morphological feature demonstrates a mixed topologic tendency, with no consensus on the bilateral occurrence or side predilection (left vs. right side) [14]. Hence, the objective of this study was to explore the frequency and pattern of distribution of radix entomolaris in mandibular first molars of a Saudi Arabian subpopulation using CBCT scans.

## 2. Materials and Methods

### 2.1. Study Design

This cross-sectional research was carried out following the recommendations for cross-sectional epidemiologic studies on root canal configuration and roots utilizing CBCT scans [27] after obtaining approval from the Dental Ethical Review Board at Qassim University, Saudi Arabia (Approval No.: EA/m-2019-3023).

### 2.2. Study Settings

The study was conducted at the dental clinics of Qassim University Medical City (QUMC) from February to May 2023.

### 2.3. Sample Size Calculation

The sample size was calculated using a Scalex sample size calculator [28]. With 5% precision, a 6.07% expected prevalence and a 95 confidence level, a minimum of 88 CBCT scans with 176 bilateral MFMs were calculated as an adequate sample size [29].

### 2.4. Participants

The CBCT scans of only Saudi national patients were included in the study by screening the patients’ files for nationality. Data acquisition was conducted in accordance with the American Association of Endodontists’ position statement [30]. The included CBCT scans were taken from 2019 to 2023 for different reasons, including the planning of treatment for endodontics, dental implants, dento-facial trauma, and orthodontic management and were obtained from the archives of the Oral Radiology Department at QUMC, Saudi Arabia. These CBCT scans were acquired using the Sirona Galileos comfort machine (Beinshiem, Germany). It had a voxel size of 160 μm and a field of view of 15 × 15 cm, and the scans were observed with GALILEOS viewer software version 1.8.

#### 2.4.1. Inclusion Criteria

Scans of Saudi national patients having bilaterally present MFMs with fully formed root apices and recorded gender/age information in patients’ files were included in the study.

#### 2.4.2. Exclusion Criteria

Scans with one/or two missing MFMs, MFMs with incomplete root apices, full- or partial-coverage prosthesis, ET, and associated radicular resorption were excluded from the study.

### 2.5. Variables

The prevalence of RE (primary outcome) and frequency of an additional canal in the main distal root, the concurrent occurrence of RE in PMFMs and permanent mandibular second molars (PMSMs), and the simultaneous presence of RE in PMFMs and a C-shaped canal in PMSMs (secondary outcomes) were determined by two endodontists with 12 years of experience (M.Q.J. and S.S.)

### 2.6. Calibration

The two evaluators were calibrated using 15 CBCT scans that were excluded from the study. Both inter-rater and intra-rater agreements were estimated for detecting and classifying RE using Cohen’s kappa test. The intra-rater reliability test was conducted after evaluating 15 CBCT scans two times at a fifteen-day interval.

### 2.7. Data Sources and Measurements

The patients’ ages and genders were also documented on the data collection sheet during data collection. The assessment method of CBCT scans comprised a distal aspect assessment of MFMs in three dimensions (axial, coronal, and sagittal) after 3D alignment of the roots’ long axis with the visualization software’s reference lines. Both evaluators were permitted to adjust tools and visualization settings (filters and noise reduction) to enhance the quality of the image. The RE (yes/no) presence was documented as suggested by Calberson and De Moor [16,17]. Moreover, the REs were classified according to Song’s classification [20].

### 2.8. Statistical Methods

The data were exported to SPPS-24 (IBM Corp, 32, Armonk, NY, USA) from the Excel sheet. The descriptive variables were documented as frequencies and percentages. The chi-square test was used to determine the association between the prevalence of RE and the gender, jaw side and age group.

## 3. Results

**Figure 1:**
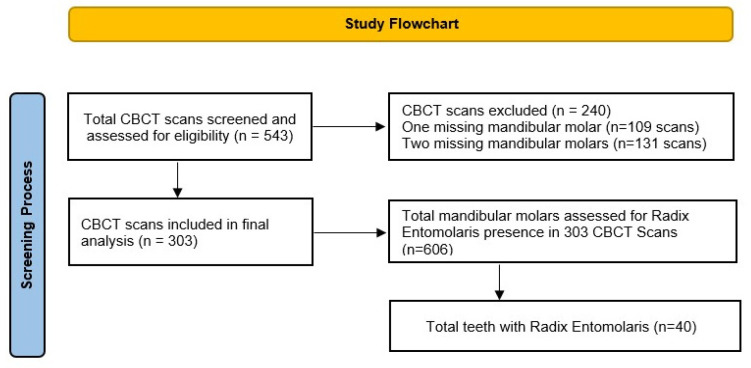
Flowchart depicting the screening process during the study.

A total of 543 CBCT scans were screened. In the final analysis, 240 CBCT scans were excluded, with one (109 scans) and two (131 scans) missing mandibular first molars. The final sample consisted of 303 CBCT scans with 606 bilaterally present mandibular first molars (Figure 1). The male to female ratio of the sample was 1.75:1, with 193 males (63.7%) and 110 females (36.3%). The mean age of the patients was 30.95 ± 11.61 years, with a range from 11 to 66 years. Absolute agreement was noted between the raters about the presence of RE and very strong agreement was noted during the classification of the RE (Cohen’s kappa: 0.98, *p* < 0.05). Moreover, intra-rater reliability was found to be 1.00 for the presence and classification of RE.

**Figure 2:**
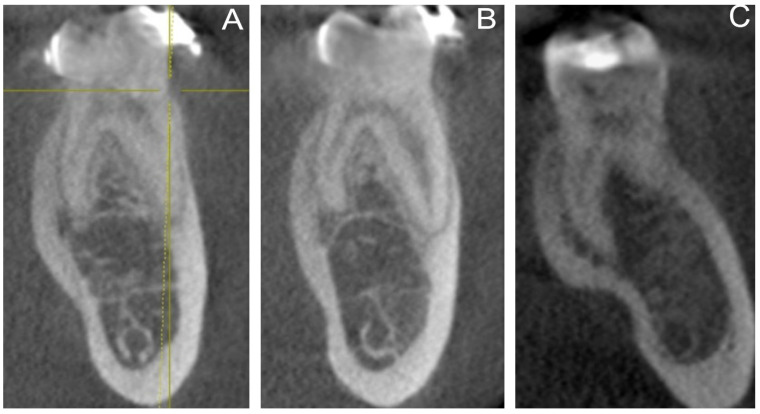
Coronal section of a CBCT image indicating the three variants of radix entomolaris according to Song et al.: (**A**) type 1, (**B**) type 2 and (**C**) type 3.

The overall prevalence of radix entomolaris (RE) was 6.6%, with no significant difference between genders (Table 1). When comparing quadrants, RE was more prevalent on the right side, but the difference was not statistically significant (Table 2). Table 3 highlights the prevalence of RE according to age groups, with the highest prevalence recorded in the 11–30 year age group and the lowest in the 51–70 year age group. A detailed analysis of the unilateral or bilateral presence of RE in relation to gender is demonstrated in Table 4. The presence of RE was either unilateral or bilateral, with comparable frequencies between genders. Type III (57.5%) was the most common variant of radix entomolaris, followed by type I (25%) and type II (17.5%) (Figure 2), whereas the small type (0%) and conical type (0%) were not identified in the study sample. Table 5 depicts the distribution of RE according to Song’s classification.

**Figure 3:**
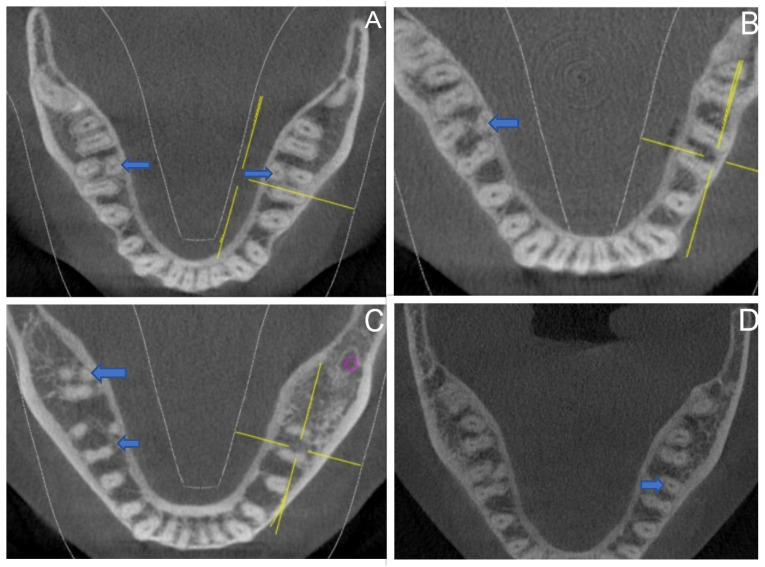
Axial sections of CBCT images indicating (blue arrows) (**A**) bilateral RE in MFM, (**B**) unilateral RE in MFM, (**C**) unilateral RE in MFM and MSM, and (**D**) an additional distal canal in the main distal root.

During the sample analysis, a few additional observations were made. When the anatomy of the main distal root was examined, only one tooth in a female patient with RE had a second distal canal in the main distal root. None of the lower second molars adjacent to the first molars with RE showed a C-shaped canal anatomy. One patient with a bilateral radix in the lower first molars also had a bilateral radix in the adjacent lower second molars. In comparison, another patient with a unilateral radix in the lower first molars had a unilateral radix in the adjacent lower second molar (Figure 3).

## 4. Discussion

The prevalence of RE is presently an issue of debate, primarily due to the discrepancies in occurrence rates amongst diverse populations. Amongst Caucasians, Africans, Eurasians, and Indians, it has been reported that RE constitutes less than 5% of the population, whereas in populations of Mongol ancestry, such as Chinese, Eskimos, and Native Americans, the frequency of RE ranges from 5% to as high as 40% [10,17,31,32].

In the present study, the overall prevalence of RE was found to be 6.6%. Our results show a slightly higher prevalence compared with the previous investigations performed in Saudi Arabia, where a proportion of 2.3% was shown by Younes et al. using extracted teeth, 5.97% was recorded by Al-Nazhan using clinical and radiographical evaluations, and 6% was recorded by Bahammam and Bahammam using extracted teeth [30,33,34]. These variations might be due to the investigation method applied. In previous studies, RE was recognized with a visual examination of extracted teeth or through periapical radiographs. However, in the present research, CBCT scans might have led to a better visualization of the roots and a higher prevalence. CBCT offers the advantage of visualizing an area from three different planes—sagittal, coronal, and axial—resulting in the elimination of the superimposition of anatomical structures, as documented [35,36]. In Asian populations of Mongol ancestry, RE is a prevalent morphological feature, with a high frequency of appearance. Research studies report a frequency exceeding 20% in some and over 30% in others [7,20,37,38]. In India, the incidence of a third root ranges from 4.5% to 13.3% [39], while in Africa, it is lower at 3.1% [40]. Conversely, in Caucasian populations, RE is considered rare, with a frequency lower than 10% [41]. This global variation in the occurrence of RE was also confirmed in a recent meta-analysis [42].

We also found that although the prevalence of RE was slightly higher in females (7.3%) than in males (6.2%), the result was not statistically significant (Table 1). Findings from other studies suggest a similar trend with no statistically significant difference in prevalence between either gender [7,9,39].

In a quadrant-wise comparison, we found that the prevalence of RE on the right side was 7.6% and on the left side it was 5.6% (Table 2). However, the result was not statistically significant. It is worth highlighting that a statistically significant association was observed in populations with a high prevalence of supernumerary roots, with a right-sided preponderance [20,43,44]. On the contrary, some researchers have documented a preference for a left-side localization [45,46]. However, there has been a relatively low frequency of reports regarding the left-sided predominance.

An age-wise comparison of the RE prevalence did not show a statistically significant difference in our study. The prevalence of RE was lowest (7.3%) among the 51–70-year-old group and highest (58.6%) among the 11–30-year-old group. Our findings are in corroboration with Talabani et al. [47], who found no statistically significant association between the age group and RE. Moreover, age-related changes in the tooth cannot interfere with the detection of RE.

The bilateral prevalence of RE in males was 2.6%, whereas in females, it was 1.98% (Table 3). These findings are in agreement with Hosseini et al. [48]. However, the present outcome falls notably below the report by Qiao et al. [49], who conducted a study on the occurrence of RE (76.87%) among MFMs in a population from Western China. This difference highlights the importance of conducting thorough clinical and radiological assessments for individuals having RE on one or both sides.

The morphological classification of RE was originally established by De Moor et al. in 2004 [17], who categorized it into three types (types I, II, and III) based on the curvature of the DL root. The classification used in this study was based on the morphological anatomy of RE by Song et al., which was a modification of De Moors’ classification [20]. This study found that type III had the highest occurrence rate at 57.5%, followed by type I at 25% and type II at 17.5%. De Moor et al. reported a prevalence rate of 61.6% for the type III morphological anatomy [17], while another study by Chen et al. only found 28.6% for type III [49], which is lower than the 57.5% found in this study. Hence, practitioners must exercise caution when treating RE, as these roots can exhibit significant curvature that can lead to potential complications in shaping the root canal, including transportation and the creation of ledges. The underdeveloped root forms, small type, and conical type were not found in the present study but have been reported in the literature, with variations existing in different geographic locations [16,40,50]. These small and conical type roots carry an increased risk of strip perforation or over-instrumentation and should be handled cautiously by the clinician [20].

In the present investigation, we have taken note of some additional observations. Firstly, out of all the scans, only one scan of a female patient had an extra canal in the distal root, making a total of five canals in her right MFM: mesiobuccal, mesiolingual, distobuccal, distolingual, and one canal in the supernumerary root situated distolingually (RE). Secondly, we investigated the presence of a C-shaped canal in the mandibular second molar adjacent to the MFM with RE. However, none of the scans investigated showed the presence of C-shaped anatomy in the mandibular second molar. Thirdly, only one scan had a bilateral RE in the MFM and mandibular second molar. Moreover, only one scan indicated the combined presence of unilateral RE in the MFM and mandibular second molar. All these root variations differ according to ethnicity, with the C-shaped canals being more common among mandibular second molars, while RE is more common among MFMs [51].

RE is a widely discussed topic due to the variations in its frequency across different populations. Ethnicity is a leading factor that influences the presence of a third root in MFM. Such a morphological variation is often present among individuals of Asian descent of Mongol ancestry [7,20]. These findings show that the prevalence of RE is a function of the demographic and geographic characteristics of the study population. Having a comprehensive knowledge of various forms of dental morphology is an essential aspect when it comes to effective endodontic treatment. Understanding this variant is critical in ensuring a successful outcome. Therefore, endodontists must thoroughly understand dental morphology to achieve an optimal result.

Diagnostic methods like sonography can be studied for the detection of RE as an alternative to CBCT. Ultrasonography (USG) was initially employed in dentistry in 1963 to evaluate tooth vitality [52]. Since that time, ultrasound imaging has been utilized in dentistry for a range of reasons, including caries detection, muscle thickness assessment, and diagnosing temporomandibular disorders [53]. In the ultrasonographic image, the alveolar bone, when in a healthy condition, exhibits full reflection and presents itself as a white surface. Similarly, the lines representing the roots of the teeth appear even whiter and are referred to as hyperechoic [54]. The use of ultrasonography in the detection of RE can be beneficial, as the sonography imaging technique may offer numerous benefits that include efficiency, cost-effectiveness, radiation-free nature with no known biological side-effects, and practical application with better patient comfort [54]. However, the efficacy of the detection of roots decreases when the thickness of the cortical plate increases, like in the mandibular posterior region [54]. Moreover, the utilization of USG for RE detection is still at a rudimentary stage.

### 4.1. Strengths and Limitations

The strength of this study lies in the use of CBCT data that are far more detailed and comprehensive than two-dimensional periapical radiographs, where the distobuccal root can easily superimpose the distobuccal root, causing an underreporting of data. Moreover, the inclusion of bilateral molars allows an analysis of bilateral prevalence that was missing in previous studies. One of the main constraints of our study was the analysis of a limited sample size confined to a particular geographic location, which restricts the external validity of the results. Moreover, the male to female ratio of the study sample (1.75:1) is not representative of the actual male to female ratio of the Saudi population (1.01:1).

### 4.2. Future Recommendations

Future CBCT-based studies with a larger sample size and male to female ratio of the sample corresponding to the actual male to female ratio of Saudi nationals is recommended, as well as the utilization of ultrasonography and Micro-CT in future research for the detection of RE.

## 5. Conclusions

To conclude, radix entomolaris is an uncommon finding among the mandibular first molars of the Saudi population without any gender and quadrant predilection. Moreover, type III is the most common variant of RE in MFMs, which may exhibit an additional distal canal on rare occasions. The findings of the current study will improve clinicians’ knowledge of the frequency of different Song’s RE types in Saudi subpopulations. Subsequently, this may contribute to the enhancement in ET outcomes.

## Figures and Tables

**Table 1:** Gender-wise prevalence of radix entomolaris and description of the total number of roots in mandibular first molars.

		Radix Entomolaris N (%)			*p*-Value *
		Absent	Present	Total	
Gender	Male	362 (93.8)	24 (6.2)	386 (63.7)	0.62
	Female	204 (92.7)	16 (7.3)	220 (36.3)	
Total		566 (93.4)	40 (6.6)	606 (100)	

* Chi-Square.

**Table 2:** Prevalence of radix entomolaris in mandibular first molars by quadrant.

		Radix Entomolaris N (%)			*p*-Value *
		Absent	Present	Total	
Tooth Type	Mandibular Left First Molar	286 (94.4)	17 (5.6)	303 (50)	0.33
	Mandibular Right First Molar	280 (92.4)	23 (7.6)	303 (50)	
Total		566 (93.4)	40 (6.6)	606 (100)	

* Chi-Square.

**Table 3:** Prevalence of radix entomolaris in mandibular first molars according to age groups.

		Radix Entomolaris N (%)			*p*-Value *
		No	Yes	Total	
Age Group	11–30	337	18	355 (58.6)	0.07
	31–50	191	16	207 (34.2)	
	51–70	38	6	44 (7.3)	
Total		566 (93.4)	40 (6.6)	606 (100)	

* Chi-Square.

**Table 4:** Radix entomolaris (distolingual root) prevalence in mandibular first molars by gender and jaw quadrant (*n* = 606).

Gender	Quadrant Side	No. of Patients	No. of Teeth	Radix Entomolaris
Male	Bilateral	8	16	16 (2.64%)
	Unilateral	8	8	8 (1.32%)
	Total	16	24	24 (3.96%)
Female	Bilateral	6	12	12 (1.98%)
	Unilateral	4	4	4 (0.66%)
	Total	10	16	16 (2.64%)
Grand Total		26	40	40 (6.6%)

**Table 5:** Song’s classification of radix entomolaris (*n* = 40).

Song’s Classification of Radix Entomolaris	Frequency (%)
Type 1	10 (25)
Type 2	7 (17.5)
Type 3	23 (57.5)
Small type	0 (0)
Conical type	0 (0)

## Data Availability

Data will be made available upon request. However, CBCT scans cannot be provided due to ethical restrictions from Deanship of Scientific Research at Qassim University, Saudi Arabia.
